# Severe rhabdomyolysis-induced acute kidney injury following concomitant use of Genvoya® (EVG/COBI/FTC/TAF) and simvastatin; a case report

**DOI:** 10.1186/s12882-019-1257-6

**Published:** 2019-02-26

**Authors:** Rita Godinho, Serge Bugnon, Terezija Gracin, James Tataw

**Affiliations:** Department of Internal Medicine, Hôpital du Jura Bernois – Moutier, Rue Beausite 49, 2740 Moutier, Switzerland

**Keywords:** Genvoya, Simvastatin, HIV, Rhabdomyolysis, Acute kidney injury

## Abstract

**Background:**

Genvoya® (elvitegravir/cobicistat/emtricitabine/tenofovir alafenamide) is a recent single regimen for the treatment of Human Immunodeficiency Virus (HIV). However, because of its complexity, it is difficult to predict drug interactions, especially when associated with HMG-CoA reductase inhibitors and/or in the setting of other comorbidities. We discuss the mechanisms of these potential drug interactions as the cause of rhabdomyolysis and acute kidney injury in the context of prior and current medication therapy with possible underlying liver and kidney dysfunction.

**Case presentation:**

We describe the case of a 54-year-old man diagnosed with HIV who developed severe rhabdomyolysis-induced anuric acute kidney injury (AKI) requiring renal replacement therapy following introduction of Genvoya® concomitantly with simvastatin, in the context of recently diagnosed hepatitis C and hepatitis A. Haemodialysis was continued over 5 weeks followed by progressive clinical and biological improvements. Five months later, a new antiretroviral regimen was started and has been well tolerated.

**Conclusion:**

Simvastatin, as well as lovastatin, because of their CYP3A4 metabolism, and to a lesser extent atorvastatin, which is only partially metabolized by CYP3A4, are the HMG-CoA reductase inhibitors with the greatest risk of drug interactions and should not be used in patients under HIV-therapy. Patients receiving HMG-CoA reductase inhibitors should be monitored regularly for the occurrence of muscular adverse effects and drug interactions should be considered with each new prescription or change in clinical status. There are many online tools that enable clinicians to rapidly check for drug interactions. We recommend the one from the University of Liverpool for patients under HIV-therapy (https://www.hiv-druginteractions.org/checker), while for patients under hepatitis C-therapy, we advise to consult http://www.hep-druginteractions.org/. This case illustrates the importance of multidisciplinary collaboration in the treatment of HIV-positive patients because of their complexity, associated comorbidities and the potential of multiple drug-drug interactions potentially exacerbated by underlying liver and/or kidney dysfunction.

## Background

The single treatment regimen Genvoya® associating elvitegravir, cobicistat, emtricitabine and tenofovir alafenamide (EVG/COBI/FTC/TAF) is a complete, simplified, efficient and safe combination pill for the treatment of Human Immunodeficiency Virus (HIV)-1 infection. It associates an integrase inhibitor (EVG), an integrase inhibitor booster (COBI) and two reverse transcriptase inhibitors (FTC and TAF). However, potential drug interactions may occur when taken together with 5-hydroxy-3-methylglutaryl-coenzyme A (HMG-CoA) reductase inhibitors. Rhabdomyolysis-induced acute kidney injury (AKI) is a potential severe complication. Suttels and al. describe a case of rhabdomyolysis-induced acute kidney injury following use of similar HIV single treatment regimen (EVG/COBI/FTC/TDF) concomitantly with pravastatin/fenofibrate [1]. To our knowledge, there is no reported case concerning Genvoya’s use and rhabdomyolysis in the literature. We describe and discuss the case of a 54-year-old patient with HIV who developed severe rhabdomyolysis-induced AKI requiring renal replacement therapy following treatment with EVG/COBI/FTC/TAF and simvastatin.

## Case presentation

A 54-year-old Caucasian man with known HIV infection for approximately 30 years had been treated with lamivudine, stavudine and indinavir since 1997. Under this therapy he was stable with an undetectable viral load. He also had dyslipidaemia which was treated with simvastatin 40 mg for many years. He presented to his primary care physician (PCP) with a 10-day history of asthenia, myalgia and jaundice. The initial laboratory revealed elevated liver enzymes (Alanine aminotransferase (ALAT) and Aspartate aminotransferase (ASAT) > 1000 U/l) and preserved kidney function (serum creatinine level of 79 μmol/l and estimated glomerular filtration rate (eGFR) of 90 ml/min/1.73 m2). He was diagnosed with acute hepatitis A and active hepatitis C genotype 1a, which was a new diagnosis. A second visit to his PCP 6 days later showed improvement of the liver enzymes. As stavudine had been withdrawn from the Swiss market, his HIV therapy was switched to Genvoya® at that time. The patient had declined referral to an HIV-specialist for confidentiality reasons.

Ten days after this medication switch, the patient presented to the emergency department (ED) with worsening myalgia and asthenia and could barely walk. He reported a reduced urine output during the prior days. There was no history of trauma, prolonged immobilization, convulsions or consumption of alcohol or illicit substances. On evaluation in the ED, the patient was slightly hypertensive at 144/84 mmHg. He presented with mucocutaneous jaundice. The cardiopulmonary status was unremarkable and the patient had no edema of the extremities. Abdominal examination revealed hepatomegaly 2 cm below the costal margin. Muscle strength was severely diminished, mostly in the axial muscles.

Initial laboratory evaluation on admission revealed elevated creatinine kinase (CK): 185190 U/I, creatinine: 553 μmol/l, phosphate: 3.03 mmol/l, potassium: 7.2 mmol/l, ASAT: 7017 U/I, ALAT: 2881 U/I, gamma-glutamyl transferase (GGT) 198 U/I, and total bilirubin: 130 μmol/l (for more details, refer to Table 1). Arterial blood gas showed a primary metabolic acidosis with a positive anion gap of 19 and appropriate respiratory compensation (Table 2). HIV-viral load was low (< 80 copies/ml) with a CD4 count > 550 cells/μl. Thyroid stimulating hormone titre was normal. Abdominal ultrasound did not show evidence of urinary or biliary tract obstruction, but it did show a homogeneous hepatosplenomegaly. No urine could be collected because of anuria.

**Table 1:** Serum parameters at the ED

Parameter	Value	Normal range
Creatine Kinase	185,190 U/l	39–308
Urea	44 mmol/l	2.5–6.4
Creatinine	553 μmol/l	66–115
Potassium	7.2 mmol/l	3.5–5
Sodium	131 mmol/l	135–145
Phosphate	3.03 mmol/l	0.84–1.52
Calcium (corrected for albumin)	2.03 mmol/l	2.10–2.55
Chloride	98 mmol/l	90–108
Aspartate aminotransferase	7017 U/l	15–37
Alanine aminotransferase	2881 U/l	16–63
Gamma-glutamyl transpeptidase	198 U/l	15–85
Total bilirubin	130 μmol/l	3–17
Prothrombin time	56%	80–110
Albumin	31 g/l	35–50
C-Reactive Protein	55 mg/l	< 3
Leukocytes	11.1 G/l	4–10
Hemoglobin	146 g/l	130–180
Hematocrit	42%	37–52
Platelets	256 G/l	150–400

**Table 2:** Blood gas analysis at the ED

Parameter	Value	Normal range
pH	7.32	7.35–7.45
Bicarbonate	14 mmol/l	22–26
pCO2	28 mmHg	35–45
Lactates	1.4 mmol/l	0.3–1.2

A diagnosis of severe rhabdomyolysis-induced AKI was made. The patient was initially treated with intravenous (IV) fluids and medical treatment for life-threatening hyperkalaemia with electrocardiographic changes. Because of refractory hyperkalaemia and metabolic acidosis, he was initiated on haemodialysis in a tertiary care setting. His antiretroviral and lipid lowering therapies were discontinued.

Intermittent haemodialysis was continued for over 5 weeks followed by progressive improvement of renal function and diuresis, serum electrolytes, liver enzymes and CK. His viral load remained low. Five months later, his creatinine had normalised to 77 μmol/l and antiretroviral therapy was restarted by an HIV-specialist with emtricitabine, tenofovir alafenamide, etravirine, ritonavir and duranavir. These were well tolerated both clinically and metabolically after several weeks. His lipid panel at the time did not warrant further treatment with a statin and he has yet to be started on treatment for hepatitis C.

## Discussion and conclusion

The patient described here presented with acute kidney injury AKIN stage III induced by severe rhabdomyolysis that was diagnosed 10 days after the introduction of Genvoya®. The main causes of rhabdomyolysis comprise crush injury, burns, infections, medications, drug use, and extreme exercise [2]. Acute kidney failure is a frequent complication [3, 4]. There are several contributing mechanisms: hypovolemia with consequent renal ischemia, tubular obstruction due to heme-pigmented casts, and tubular lesions due to free iron [4]. Our patient had no history of recent trauma, prolonged immobilization, convulsions or consumption of alcohol or illicit substances. Infections other than HIV and hepatitis A and C were ruled out during the diagnostic evaluation. HIV infection itself can lead to rhabdomyolysis during the acute infection stage [3], as well as, although rarely, HIV-associated myopathy [5, 6]. These were unlikely in this case given the undetectable HIV viral load and a high CD4 count.

The Naranjo Adverse Drug Reaction Probability Score [7], which allows for evaluation of the probability that a secondary effect is due to a given medication, was 5, indicating that the rhabdomyolysis in this case could probably be attributed to an adverse response to medication. In addition to the newly prescribed Genvoya®, the patient was taking simvastatin. He had been taking simvastatin without adverse effects for many years.

Statins (HMG-CoA reductase inhibitors) act as direct myotoxins [8], although they themselves are rarely responsible for massive rhabdomyolysis. Interactions between statins and other medications that interfere with their metabolism can however trigger adverse effects. Inhibitors of HMG-CoA reductase are widely prescribed in the general population to treat hypercholesterolemia and to prevent cardiovascular morbidity and mortality. They are increasingly prescribed for the HIV-positive patients due to high prevalence of dyslipidaemia in this population resulting from HIV infection itself and the adverse effects of the antiretroviral treatment [9]. While they are generally well-tolerated, various afflictions of skeletal muscles can occur, ranging from myalgia to severe rhabdomyolysis [10]. The various statins have different pharmacokinetic profiles.

Simvastatin, the statin prescribed for our patient, is one of the three metabolized by cytochrome P450 3A4 (CYP3A4). Its potential for interaction with potent inhibitors of CYP3A, such as COBI, is one of the highest. COBI, one of the four constituents of Genvoya®, lacks intrinsic anti-HIV activity. It is used as a booster of EVG, allowing for higher plasma concentrations of the latter with lower administered doses, thereby reducing secondary effects [10] (see Table 3). Several case-reports have established a clear relationship between the coadministration of pharmacokinetic boosters with simvastatin and the occurrence of a rhabdomyolysis, with evidence for a significant increase in the area under the curve (AUC) and the maximal concentration of statin [10]. The patient had been on simvastatin for many years, associated with the triple antiretroviral therapy lamivudin, stavudin, and indinavir. Ten days after substitution of the latter with Genvoya®, which includes COBI, the patient developed severe rhabdomyolysis associated with anuric AKI. It is highly plausible that the COBI-simvastatin combination played a pivotal role in this clinical presentation. Current guidelines indeed argue against combination of these two drugs. Such a contra-indication may extend to the coadministration of simvastatin with other inhibitors of CYP3A. The risk of statins interacting with other medications, and consequently the risk of myopathy, is proportional to the dose of the statin.

**Table 3:** Select CYP3A4 inhibitors and substrates among the components of Genvoya® and simvastatine

Inhibitors CYP3A4	Substrates CYP3A4
Cobicistat	Elvitegravir Simvastatine

Other factors may also have contributed to our patient’s rhabdomyolysis. The treatment with Genvoya® was introduced in the presence of a recently diagnosed liver disease from hepatitis A and C, with abnormal transaminase levels. At this time, liver function was not fully tested. In this context, a certain degree of hepatocellular dysfunction can nonetheless be assumed that, as a result of the reduced metabolism, could have led to a higher statin plasma concentration. Similarly, the prior triple therapy of the patient included indinavir, a protease inhibitor metabolized primarily by CYP3A4, for which it is also a potent inhibitor. Although this triple therapy was tolerated very well over the years in association with simvastatin, it is possible that the recent liver disease increased the plasma concentration of indinavir, as a result of a decrease in its metabolism, leading to an increase in plasma simvastatin concentration. The fact that our patient suffered from myalgia several days prior to the introduction of Genvoya® could indicate that rhabdomyolysis may have begun in this context. Moreover, the EVG present in the Genvoya® is also metabolized in the liver by cytochrome CYP3A4. Due to a competitive effect, this may have increased the concentration of simvastatin, thereby promoting the occurrence of the rhabdomyolysis.

According to Lima et al. [11], underlying kidney dysfunction is a risk factor for the development of a rhabdomyolysis from statins. Tenofovir, present as the prodrug TAF in Genvoya®, is known for its potential nephrotoxicity [12]. It is a potent reverse transcriptase nucleotide inhibitor that has to be administered as a prodrug due to its poor pharmacokinetic and pharmacodynamic properties [13]. The first prodrug of tenofovir to be commercially available, tenofovir disoproxyl fumarate (TDF) was associated with cases of Fanconi syndrome, acute kidney failure, and nephrogenic diabetes insipidus [14]. HIV/hepatitis C co-infection has been identified as a risk factor in this setting [15, 16]. TAF was developed with the aim of having a better renal (and bone) safety profile. Although phase III trials have revealed a better safety profile compared to TDF [17–19], the possibility of nephrotoxicity due to tenofovir, or even subtle underlying renal dysfunction due to HIV itself could have increased the risk for severe AKI in our patient. Nonetheless, the fact that TAF is part of the new pentatherapy of the patient, and that it is currently well tolerated speaks against this hypothesis.

Medication interactions are avoidable. AKI is a severe complication and even after recovery is associated with increased long term mortality and risk of chronic kidney disease [20]. This case illustrates the importance of vigilance when prescribing new medications, especially in multi-morbid patients. Both prescribing clinicians and pharmacists should be aware of potential interactions, and use of medication compendia and interaction checkers such as the online tool from the University of Liverpool for patients under HIV-therapy (https://www.hiv-druginteractions.org/checker) or http://www.hep-druginteractions.org/ for patients under hepatitis C-therapy which are easily available online should become routine in the clinic.

It is important to recall that drug interactions associated with CYP3A4 metabolism occur with a wide range of medications including commonly used drugs such as, among many others, amiodarone, calcium-channel blockers, azole antifungals and macrolides antibiotics (Table 4). In a FDA database review, the majority (58%) of cases of statin-induced rhabdomyolysis occurred through the association with another drug interfering with statin metabolism through CYP3A4 [21, 22].

**Table 4:** Substrates, inducers and inhibitors of the cytochrome P450 enzymatic pathways [21, 23]

CYP3A4 substrates	Inducers	Inhibitors
Statins (simvastatin, atorvastatin, lovastatin) Elvitegravir Hormonal contraceptives Corticosteroids (budesonide, fluticasone, triamcinolone) Direct Xa inhibitors (rivaroxaban, apixaban) Dihydropyridine calcium channel blockers (nifedipine, felodipine) Antiarrhythmic (amiodarone, propafenone, flecainide) Benzodiazepines (midazolam, alprazolam, triazolam)	Anti-epileptic drugs (carbamazepine, phenytoin, phenobarbital) Rifabutin, Rifampin Dexamethasone Omeprazole Non-nucleoside reverse transcriptase inhibitors (efavirenz, nevirapine, etravirine)	Azoles antifungals (fluconazole, itraconazole, ketoconazole) Macrolides (erythromycin, clarithromycin, azithromycin) Antidepressants (tricyclic, venlafaxine, fluoxetine, sertraline) Calcineurin inhibitors (cyclosporine, tacrolimus) Nondihydropyridine calcium channel blockers (verapamil, diltiazem) Amiodarone Protease inhibitors (indinavir, amprenavir, nelfinavir, saquinavir, atazanavir, ritonavir) Cobicistat Grapefruit juice

This case reveals the complexity of the treatment of HIV-positive patients who are at risk of multiple potential drug interactions, especially when statins are associated with fixed anti-retroviral drug combinations and in the setting of potential liver and/or kidney dysfunction. Simvastatin, lovastatin and, to a lesser extent atorvastatin, are the HMG-CoA reductase inhibitors with the greatest risk of drug interactions [9]. They should not be used in patients under HIV-therapy. Heightened vigilance for potential drug interactions is essential when treating patients with HIV and multidisciplinary collaboration is advised.

## Abbreviations

AKI: Acute kidney injury
ALAT: Alanine aminotransferase
ASAT: Aspartate aminotransferase
AUC: Area under the curve
CK: Creatine kinasis
COBI: Cobicistat
CYP3A4: Cytochrome P450 3A4
ED: Emergency department
eGFR: estimated Glomerular filtration rate
EVG: Elvitegravir
FTC: Emtricitabine
GGT: Gamma-glutamyl transferase
HIV: Human immunodeficiency virus
HMG-CoA: 5-hydroxy-3-methylglutaryl-coenzyme A
IV: Intravenous
PCP: Primary care physician
TAF: Tenofovir alafenamide
TDF: tenofovir disoproxyl fumarate

## References

[1] Suttels V, Florence E, Leys J, Vekemans M, Van den Ende J, Vlieghe E (2015). A 68-year old male presenting with rhabdomyolysis-associated acute kidney injury following concomitant use of elvitegravir/cobicistat/emtricitabine/tenofovir disoproxil fumarate and pravastatin/fenofibrate: a case report. J Med Case Rep..

[2] Spiegel LR, Schrier PB, Shah HH (2013). Severe recurrent rhabdomyolysis-induced acute kidney injury in a HIV-infected patient on antiretroviral therapy. Ren Fail..

[3] Gabow PA, Kaehny WDKS (1982). The spectrum of rhabdomyolysis. Med..

[4] Miller ML. Clinical manifestations and diagnosis of rhabdomyolysis. UpToDate, Wolters Kluwer. 2017. https://www.uptodate.com. Accessed 19 Sept 2017.

[5] Robinson-Papp J, Simpson DM (2009). Neuromuscular diseases associated with HIV-1 infection. Muscle Nerve..

[6] Koubar SH, Estrella MM, Warrier R, Moore RD, Lucas GM, Atta MG (2017). Rhabdomyolysis in an HIV cohort: epidemiology. causes and outcomes. BMC Nephrol..

[7] Naranjo CA, Busto U, Sellers EM, Sandor P, Ruiz I, Roberts EA (1981). A method for estimating the probability of adverse drug reactions. Clin Pharmacol Ther..

[8] Miller ML. Causes of rhabdomyolysis. UpToDate, Wolters Kluwer. 2017. vhttps://www.uptodate.com. Accessed 19 Sept 2017.

[9] Penzak SR, Chuck SKSG (2000). Safety and efficacy of HMG-CoA reductase inhibitors for treatment of hyperlipidemia in patients with HIV infection. Pharmacotherapy..

[10] Chauvin B, Drouot S, Barrail-Tran A, Taburet A-M (2013). Drug–Drug Interactions Between HMG-CoA Reductase Inhibitors (Statins) and Antiviral Protease Inhibitors. Clin Pharmacokinet..

[11] Lima R, Junior G, Liborio A, Daher E (2008). Acute Kidney Injury due to Rhabdomyolysis. Saudi J Kidney Dis Transpl..

[12] Scherzer R, Estrella M, Li Y, Choi AI, Deeks SG, Grunfeld C (2012). Association of tenofovir exposure with kidney disease risk in HIV infection. AIDS..

[13] Sauvage A-S, Darcis G, Moutschen M (2016). Actualité thérapeutique dans le VIH : le ténofovir alafénamide. Rev Med Suisse..

[14] Tourret J, Deray G, Isnard-Bagnis C (2013). Tenofovir Effect on the Kidneys of HIV-Infected Patients: A Double-Edged Sword?. J Am Soc Nephrol..

[15] Campos P, Ortiz A, Soto K (2016). HIV and kidney diseases: 35 years of history and consequences. Clin Kidney J..

[16] Fernandez-Fernandez B, Montoya-Ferrer A, Sanz AB, Sanchez-Niño MD, Izquierdo MC, Poveda J (2011). Tenofovir Nephrotoxicity: 2011 Update. AIDS Res Treat..

[17] Sax PE, Wohl D, Yin MT, Post F, DeJesus E, Saag M (2015). Tenofovir alafenamide versus tenofovir disoproxil fumarate, coformulated with elvitegravir, cobicistat, and emtricitabine, for initial treatment of HIV-1 infection: two randomised, double-blind, phase 3, non-inferiority trials. Lancet..

[18] Raffi F, Orkin C, Clarke A, Slama L, Gallant J, Daar E (2017). Brief Report: Long-Term (96-Week) Efficacy and Safety After Switching From Tenofovir Disoproxil Fumarate to Tenofovir Alafenamide in HIV-Infected, Virologically Suppressed Adults. JAIDS J Acquir Immune Defic Syndr..

[19] Post FA, Yazdanpanah Y, Schembri G, Lazzarin A, Reynes J, Maggiolo F (2017). Efficacy and safety of emtricitabine/tenofovir alafenamide (FTC/TAF) vs. emtricitabine/tenofovir disoproxil fumarate (FTC/TDF) as a backbone for treatment of HIV-1 infection in virologically suppressed adults: subgroup analysis by third agent of a randomi. HIV Clin Trials..

[20] Doyle JF, Forni LG (2016). Acute kidney injury: short-term and long-term effects. Crit Care..

[21] Bellosta S. Safety of Statins: Focus on Clinical Pharmacokinetics and Drug Interactions. Circulation [Internet]. 2004 Jun 15;109(23_suppl_1):III-50-III-57. Available from: http://circ.ahajournals.org/cgi/doi/10.1161/01.CIR.0000131519.15067.1f 10.1161/01.CIR.0000131519.15067.1f 15198967

[22] Marot A, Morelle J, Chouinard VA, Jadoul M, Lambert M, Demoulin N (2011). Concomitant use of simvastatin and amiodarone resulting in severe rhabdomyolysis: A case report and review of the literature. Acta Clin Belg..

[23] Stolbach A, Paziana K, Heverling H, Pham P. A Review of the Toxicity of HIV Medications II: Interactions with Drugs and Complementary and Alternative Medicine Products. J Med Toxicol [Internet]. 2015 Sep 3;11(3):326–341. Available from: http://link.springer.com/10.1007/s13181-015-0465-0 10.1007/s13181-015-0465-0 PMC4547966 26036354

